# Traumatic Intracranial Hemorrhage Correlates with Preinjury Brain Atrophy, but Not with Antithrombotic Agent Use: A Retrospective Study

**DOI:** 10.1371/journal.pone.0109473

**Published:** 2014-10-03

**Authors:** C. Michael Dunham, David A. Hoffman, Gregory S. Huang, Laurel A. Omert, David J. Gemmel, Renee Merrell

**Affiliations:** 1 Trauma/Critical Care Services, St. Elizabeth Health Center, Youngstown, Ohio, United States of America; 2 Division of Cardiology, St. Elizabeth Health Center, Youngstown, Ohio, United States of America; 3 CSL Behring, King of Prussia, Pennsylvania, United States of America; 4 Medical Education and Statistics, St. Elizabeth Health Center, Youngstown, Ohio, United States of America; University of Wuerzburg, Germany

## Abstract

**Background:**

The impact of antithrombotic agents (warfarin, clopidogrel, ASA) on traumatic brain injury outcomes is highly controversial. Although cerebral atrophy is speculated as a risk for acute intracranial hemorrhage, there is no objective literature evidence.

**Materials and Methods:**

This is a retrospective, consecutive investigation of patients with signs of external head trauma and age ≥60 years. Outcomes were correlated with antithrombotic-agent status, coagulation test results, admission neurologic function, and CT-based cerebral atrophy dimensions.

**Results:**

Of 198 consecutive patients, 36% were antithrombotic-negative and 64% antithrombotic-positive. ASA patients had higher arachidonic acid inhibition (p = 0.04) and warfarin patients had higher INR (p<0.001), compared to antithrombotic-negative patients. Antithrombotic-positive intracranial hemorrhage rate (38.9%) was similar to the antithrombotic-negative rate (31.9%; p = 0.3285). Coagulopathy was not present on the ten standard coagulation, thromboelastography, and platelet mapping tests with intracranial hemorrhage and results were similar to those without hemorrhage (p≥0.1354). Hemorrhagic-neurologic complication (intracranial hemorrhage progression, need for craniotomy, neurologic deterioration, or death) rates were similar for antithrombotic-negative (6.9%) and antithrombotic-positive (8.7%; p = 0.6574) patients. The hemorrhagic-neurologic complication rate was increased when admission major neurologic dysfunction was present (63.2% versus 2.2%; RR = 28.3; p<0.001). Age correlated inversely with brain parenchymal width (p<0.001) and positively with lateral ventricular width (p = 0.047) and cortical atrophy (p<0.001). Intracranial hemorrhage correlated with cortical atrophy (p<0.001) and ventricular width (p<0.001).

**Conclusions:**

Intracranial hemorrhage is not associated with antithrombotic agent use. Intracranial hemorrhage patients have no demonstrable coagulopathy. The association of preinjury brain atrophy with acute intracranial hemorrhage is a novel finding. Contrary to antithrombotic agent status, admission neurologic abnormality is a predictor of adverse post-admission outcomes. Study findings indicate that effective hemostasis is maintained with antithrombotic therapy.

## Introduction

Among the elderly in the United States, there are 155,000 cases of traumatic brain injury annually, leading to 12,000 deaths [1]. Chronic oral anticoagulation or antiplatelet agents have been used with increasing frequency for reducing the risk of venous thromboembolism, preventing clot formation and stroke development with atrial fibrillation, and reducing coronary artery thrombosis in patients with atherosclerosis and cardiac stents [2], [3]. The prevalence of warfarin use in the United States is unknown, but the Food and Drug Administration estimates that more than 31 million prescriptions for warfarin were written in 2004 [4]. A publication by Dossett et al. indicates that warfarin use is common among injured patients and its prevalence has increased each year since 2002 [5].

Numerous studies assessing the impact of antithrombotic (AT) agents on traumatic brain injury reveal conflicting results. Some investigations indicate that AT drugs are associated with increased brain-injury mortality [6]–[12], while others suggest that this is not the case [13]–[19]. Although certain studies demonstrate that AT medications increase intracranial hemorrhage (ICH) rates [6], [7], [10], [12], [15], others differ [8], [19]. Also at odds are findings that AT agents are associated with increased ICH progression [16], while some studies observed contrary findings [19], [20].

Recent meta-analyses objectively summarize the controversy that exists regarding the impact of AT-agents on traumatic brain injury (TBI) outcomes. The meta-analysis performed for ASA and the separate meta-analysis for clopidogrel demonstrated that the tests for data result heterogeneity were statistically significant and the overall mortality rates, with and without the respective AT-agent, were not statistically different [21]. A separate meta-analysis for warfarin also showed that the statistic for data results heterogeneity was significant [22]. A significant meta-analysis test for heterogeneity indicates that there was substantial variance among the studies, relative to AT-positive and AT-negative mortality rate differences. The significant test for heterogeneity in each of the 3 meta-analyses (ASA, clopidogrel, and warfarin) clearly demonstrates that the impact of these AT-agents on TBI mortality is controversial.

Several publications have included comments suggesting that preinjury cerebral atrophy predisposes to acute ICH [23]–[27]. The relevant statement in each publication had a referenced citation. However, a review of the referenced material failed to describe any objective evidence that preinjury cerebral atrophy engenders acute ICH.

Due to the controversial results in the literature regarding the impact of AT agents on brain-injury outcomes, the authors undertook a retrospective investigation of consecutive patients to further examine this issue. First, we hypothesized AT-positive patients would not have an increased hemorrhage rate, higher rate of hemorrhagic complications, or worse neurologic outcomes. Second, we hypothesized that admission neurological function would better predict brain injury outcomes, than would a history of AT agent use. Third, we conjectured that intracranial hemorrhage would correlate with the degree of preinjury brain atrophy.

## Materials and Methods

This study was approved by the Humility of Mary Health Partners Institutional Review Board for Human Investigations. The Institutional Review Board waived the need for written informed consent from the patients, due to the retrospective nature of the investigation. Inclusion criteria utilized: age ≥60 years, fall from standing height or motor vehicular crash, physical evidence for head trauma (facial fracture, skull fracture, scalp soft tissue injury, facial soft tissue injury, or cervical spine injury), and trauma center admission. An Abbreviated Injury Scale score (AIS) for each body region was determined by the trauma registrar after receiving input from radiographic reports, operative records, Advanced Practice Nurses, and attending trauma surgeons. Regional AIS values were stored in the trauma registry and a standardized process was used to compute Injury Severity Scores.

### Patient outcomes

Admission major neurologic dysfunction was defined as patients with Glasgow Coma Score 3–12 and/or the presence of speech aphasia or severe agitation. Patients who were prescribed ASA, clopidogrel, warfarin, or rivaroxaban preinjury were classified as AT-positive. Those who were not prescribed these agents were classified as AT-negative. ICH was determined to be absent or present and subcategorized as creating or not creating brain compression. ICH complications were the need for craniotomy or ICH progression. Neurologic complications were hospital death, transfer to hospice, or a decrease in discharge Glasgow Coma Score ≥ three points below the admission value.

The Glasgow Outcome Score (GOS) was determined at 3 months following trauma center discharge. The electronic medical record was reviewed for patient follow-up, which included re-hospitalization, clinic and office visits, and telephone conversations. The 3-month GOS was dichotomized into a good-outcome (moderate or minimal-to-no disability) or bad-outcome (death, vegetative state, or severe disability). When the data were insufficient to categorize a patient, the GOS category was considered unknown.

### Coagulation profile

Standard coagulation testing results included partial thromboplastin time, International Normalized Ratio (INR), and platelet count. Thromboelastography results included R-Time, K-Time, alpha-angle, maximum amplitude, and Coagulation Index. Thromboelastography platelet mapping included arachidonic acid and adenosine diphosphate percent inhibition. A coagulation intervention included the administration of platelets, desmopressin, plasma, and/or intravenous vitamin K. Policy dictated that a Trauma Service Advanced Practice Nurse must document, within 48 hours, the use of all preinjury AT agents for each patient admitted to the trauma center. Standard coagulation, thromboelastography, and platelet mapping tests were performed by the Central Laboratory Services.

### Preinjury brain atrophy

Brain CT reports were reviewed to determine how often the radiologist commented on the presence of brain atrophy. Axial views of the brain CT were reviewed to locate and measure the maximal transverse width of the left and right lateral ventricular bodies. A brain parenchymal width (right and left transverse distance from cortical surface to ipsilateral ventricular margin) was measured at the axial level of the maximal lateral ventricular body width. At the level of the maximal lateral ventricular body width, the cortical sulci were scored to assess cortical atrophy [28]. Cortical atrophy was considered insignificant with a score of 0 or 1 and was considered present with a score of 2 or 3.

### Statistical analysis

Data were entered into a Microsoft Excel 2010 spreadsheet and imported into a SAS System for Windows, release 9.2 (SAS Institute Inc., Cary, NC, USA), for statistical analyses. For continuous variable cohort data, standard deviation was used to complement the mean. Non-parametric analysis was used to compare continuous data results between two groups. Fischer's exact testing was used to assess the relationship of two dichotomous variables. Multivariate logistic regression analysis was performed to assess independent variable relationships with a dependent variable that was dichotomous.

## Results

### Patient traits

In this consecutive series, there were 198 patients with an age of 78.4±10 years. All patients had an admission brain CT scan and 71 of 72 with ICH had a repeat brain CT (one rapidly progressed to brain death). Patient injury traits are summarized in **Table 1** and preexisting medical conditions according to AT status are in **Table 2**.

**Table 1:** Patient Traits and Outcomes.

	Number	Percent
**Number**	198	100%
**Admission Glasgow Coma Score 3–12**	15	7.6%
**Admission Major Neurologic Dysfunction**	19	9.6%
**Fall from Standing Height**	162	81.8%
**Motor Vehicular Collision**	36	18.2%
**Antithrombotic-Negative**	72	36.4%
**Antithrombotic-Positive**	126	63.6%
**ASA**	89	45.0%
**Clopidogrel**	18	9.1%
**ASA and/or Clopidogrel (no anticoagulant)**	76	38.4%
**Warfarin**	46	23.2%
**Rivaroxaban**	4	2.0%
**Preinjury Brain Atrophy**	98	49.5%
**Intracranial Hemorrhage**	72	36.4%
**Intracranial Hemorrhage with Brain Compression**	12	6.1%
**Intracranial Hemorrhage Complication**	8	4.0%
**Neurologic Complication**	13	6.6%
**Intracranial Hemorrhage-Neurologic Complication**	16	8.1%

**Table 2:** Pre-existing Medical Conditions by Antithrombotic Status.

Condition	Antithrombotic (−)	Antithrombotic (+)	P-value
**Cerebrovascular Accident**	4.3%	6.5%	0.5227
**Cardiac Disease**	20.0%	58.5%	<0.001
**Diabetes Mellitus**	24.3%	34.2%	0.1528
**Dementia**	22.9%	19.5%	0.5815
**Hypertension**	64.3%	79.7%	0.0190
**Psychiatric Disorder**	15.7%	13.8%	0.7196
**Pulmonary Disease**	12.9%	17.9%	0.3603
**Smoker (Active)**	15.7%	4.1%	0.0048

### Coagulation profile

Of the 198 patients, standard coagulation testing was partial thromboplastin time in 166 (83.8%), INR in 184 (92.9%), and platelet count in 187 (94.4%). Of the 72 AT-negative patients, 21 (29.2%) had thromboelastography and 14 (19.4%) platelet mapping. Of the 126 AT-positive patients, 97 (77.0%) had thromboelastography and 83 (65.9%) platelet mapping. The coagulation profile, according to AT status, is displayed in **Table 3**. ASA patients had a higher arachidonic acid percent inhibition and lower Coagulation Index, compared to the AT-negative group. Warfarin patients had higher INR and R-Time values and lower Coagulation Index, compared to the AT-negative group. Of the 126 patients in the AT-positive group, ASA and/or warfarin patients accounted for 121 (96.0%).

**Table 3:** Coagulation Profile According to Pre-Injury Antithrombotic Status.

	AT (−)	Aspirin	Clopidogrel	Warfarin	Rivaroxaban
**Number**	72	89	18	46	4
**International Normalized Ratio**	1.2±0.2	1.4±0.8	1.3±0.7	2.8±0.9**	1.5±0.6
**Partial Thromboplastin Time**	29±5	31±7	28±7	38±8**	29±6
**Platelet Count**	211±64	215±75	215±64	199±60	215±71
**R-Time**	4.1±1.3	5.5±2.1	4.8±1.3	6.4±2.2**	3.8±1.8
**K-Time**	1.2±0.4	1.4±0.6	1.3±0.3	1.5±0.5	1.1±0.4
**Alpha Angle**	72±5	69±9	71±4	69±7	64±22
**Maximum Amplitude**	65±6	67±5	66±5	67±4	65±9
**Coagulation Index**	2.6±1.6	1.5±2.4**	2.2±1.3	1.0±2.2**	2.1±3.4
**Arachidonic Acid % inhibition**	41±42	62±31**	52±37	35±39	29±50
**Adenosine Diphosphate % inhibition**	44±23	48±33	49±30	40±26	50±27

AT (−), Antithrombotic-negative;

**: P<0.05, compared to Antithrombotic-negative group

### Preinjury brain atrophy

The CT report included a statement that age-related changes were present in 170 (87.4%). Of the 12 with ICH mass effect, seven had a brain CT within the prior 12 months and were scored. Scoring was not performed for the other five. Because one Glasgow Coma Score 15 patient had a normal brain CT at a referring facility, the scan was not archived and available for scoring. Thus, 192 of the 198 (97.0%) patients underwent CT grading to evaluate evidence of atrophy. Dimension results were: ventricular diameter 31.4±5.6 mm; brain diameter 90.6±8.3 mm; and cortical atrophy (sulcus score of 2 or 3) in 31.8%. Ventricular width was higher in patients with cortical atrophy (34.1±4.7 mm), when compared to no atrophy (30.1±5.5 mm; p<0.001). Composite brain atrophy (cortical atrophy or ventricular width>30 mm) was found in 51.0% (n = 98). Increasing age had an inverse correlation with brain width (p<0.001) and positive associations with ventricular width (p = 0.0465) and ventricular width ÷ brain width (p = 0.0014). Age was higher in patients with cortical atrophy (83.0±8.2 years), compared to no atrophy (76.2±10.1 years; p<0.001).

### Intracranial hemorrhage correlations

The ICH rates, according to each head trauma risk condition, were cervical spine injury 12.5% (3/24), facial fracture 21.6% (8/37), skull fracture 31.6% (6/19), facial soft tissue injury 43.5% (10/23), and scalp soft tissue injury 35.6% (53/149). The frequency of each head trauma ICH risk condition, by AT-negative and AT-positive grouping, is depicted in **Table 4**; risk conditions for the two groups are clinically and statistically similar.

**Table 4:** Head Trauma ICH Risk Conditions by Antithrombotic Status.

	AT-negative	AT-positive	P-value
**Number**	**72**	**126**	
**Cervical spine injury**	11 (15.3%)	13 (10.3%)	0.3663
**Facial fracture**	17 (23.6%)	20 (15.9%)	0.1895
**Skull fracture**	10 (13.9%)	9 (7.1%)	0.1371
**Facial soft tissue injury**	9 (12.5%)	14 (11.1%)	0.8193
**Scalp soft tissue injury**	54 (75.0%)	95 (75.4%)	0.9504

ICH, intracranial hemorrhage; AT, antithrombotic.

Of the 198 patients, ICH was present in 72 (36.4%) and caused major brain compression in 12 (6.1%). The ICH rate was higher with cortical atrophy (60.7%), compared to no atrophy (22.9%; p<0.001; OR = 5.2). Ventricular width was higher with ICH (33.7±5.4), compared to no ICH (30.1±5.3; p<0.001). Ventricular width ÷ brain width was higher with ICH (37.7±7.1%), when compared to no ICH (33.5±6.9%; p<0.001). ICH rate was higher with ventricular width>30 mm (57.0%), versus width ≤30 mm (19.5%; p<0.001; OR = 5.5). The ICH rate was higher with composite brain atrophy (54.1%), compared to no brain atrophy (14.9%; p<0.001; OR = 6.7).

The AT-positive ICH rate (38.9% [49/126]; [ASA - 42.7%; clopidogrel - 50.0%; warfarin or rivaroxaban - 28.0%]) was similar to the AT-negative ICH rate (31.9% [23/72]; p = 0.3285; Lambda proportional reduction in error  = 0.000). ICH was similar for warfarin-positive (28.3% [13/46]) and AT negative (31.9% [23/72]; p = 0.8378; lambda proportional reduction in error  = 0.0000) patients. ICH is insignificantly higher for platelet inhibitor-positive (46.1% [35/76]), compared to AT negative (31.9% [23/72]; p = 0.0932; lambda proportional reduction in error  = 0.1111) patients. Relative to the ICH that created brain deformation, the AT-positive rate (7.1%) was similar to the AT-negative rate (4.2%; p = 0.5415). The ISS and highest regional AIS values, according to AT-positive and AT-negative status, are displayed in **Table 5**. The Injury Severity Scores and AIS values for the AT-positive and AT-negative patients were not statistically different. Head AIS scores for ICH-positive patients were similar according to AT-negative (3.6±0.8) and AT-positive (3.4±1.1; p = 0.4265) status.

**Table 5:** Injury Severity Score and AIS Values by Antithrombotic Status.

	AT-negative	AT-positive	P-value
**Number**	72	126	
**Injury Severity Score**	11.9±7.6	10.6±7.5	0.2391
**Head AIS**	1.9±1.6	1.9±1.6	0.9452
**Head AIS ≥3**	27 (37.5%)	41 (32.5%)	0.5347
**Chest AIS ≥1**	10 (13.9%)	19 (15.1%)	0.8197
**Abdominal-pelvis AIS ≥1**	1 (1.4%)	5 (4.0%)	0.4196
**Extremity-pelvic AIS ≥1**	17 (23.6%)	31 (24.6%)	0.8755

AIS, Abbreviated Injury Scale score; AT, antithrombotic.

Standard coagulation, thromboelastography, and platelet mapping results demonstrated no coagulopathy in ICH-positive patients and were similar to the ICH -negative patients (**Table 6**). Of the 89 patients receiving preinjury ASA, the ICH-negative arachidonic acid percent inhibition (62±31) and Coagulation Index (1.9±1.8) (n = 51) were similar to the ICH-positive arachidonic acid percent inhibition (62±32) and Coagulation Index (1.2±3.0) (n = 38) (p≥0.2657). For the 18 patients receiving preinjury clopidogrel, the ICH-negative adenosine diphosphate percent inhibition (49±34) and Coagulation Index (2.6±1.2) (n = 9) were similar to the ICH-positive adenosine diphosphate percent inhibition (49±26) and Coagulation Index (1.6±0.4) (n = 9) (p≥0.1043). In the 46 patients receiving preinjury warfarin, the ICH-negative INR (2.8±1.0), R-Time (6.1±2.2), and K-Time (1.5±0.6) (n = 33) were similar to the ICH-positive INR (2.8±0.5), R-Time (7.1±2.1), and K-Time (1.5±0.3) (n = 13) (p≥0.2829).

**Table 6:** Coagulation Profile According to Intracranial Hemorrhage Status.

	Hemorrhage (−)	Hemorrhage (+)	P-Value
**Number**	**126**	**72**	—
**International Normalized Ratio**	1.6±0.9	1.4±0.7	0.1354
**Partial Thromboplastin Time**	31±6	32±9	0.5472
**Platelet count**	207±61	213±79	0.5657
**R-Time**	5.2±1.9	5.6±2.3	0.3840
**K-Time**	1.3±0.4	1.5±0.7	0.2310
**Alpha-Angle**	70±8	70±8	0.9950
**Maximum Amplitude**	67±4	66±7	0.3759
**Coagulation Index**	1.8±2.0	1.4±2.7	0.3884
**Arachidonic Acid % inhibition**	46±38	49±37	0.7090
**Adenosine Diphosphate % inhibition**	44±31	48±32	0.5047

(−), negative; (+), positive.

Composite brain atrophy was more common for AT-positive (56.9% [70/123]) compared to AT negative (40.6% [28/69]; p = 0.0355) patients. Brain atrophy occurrence was 56.8% for platelet inhibitor-positive patients and 55.6% for warfarin-positive patients. Multivariate analysis showed that ICH correlated with composite brain atrophy (p<0.0001), but not AT agent status (p = 0.9293) (n = 192 AT-positive or AT-negative patients). ICH correlated with composite brain atrophy (p<0.0001), but not platelet inhibitor agent status (p = 0.3205) (n = 143 AT-negative or platelet inhibitor-positive patients). ICH correlated with composite brain atrophy (p<0.0001), but not warfarin status (p = 0.2733) (n = 114 AT-negative or warfarin-positive patients). ICH had an independent association with composite brain atrophy (p<0.001) and admission major neurologic dysfunction (p<0.001), but not AT status (p = 0.9774) or age (p = 0.8566).

### Admission neurologic function and complication correlations

Admission major neurologic dysfunction rates were comparable for the AT-negative (8.3%) and AT-positive (10.3%; p = 0.6484) patients. The admission major neurologic dysfunction rate was greater for ICH-positive patients (20.8%) versus ICH-negative patients (3.2%; OR = 8.0; p<0.001). Of the 198 patients, 16 (8.1%) had an ICH-neurologic complication. Of those 16, 12 (75.0%) died or were transferred to hospice, with an age of 79.1±9.4 (65–95). ICH expansion occurred or a craniotomy was needed in 3 of the 4 survivors. The other survivor was GCS 15 on admission and GCS 12 at hospital discharge.

The ICH-neurologic complication rate was similar for AT-positive patients (8.7% [11/126]) and AT-negative patients (6.9% [5/72]; p = 0.6574). The ICH-neurologic complication rate was similar for platelet inhibitor-positive (7.9% [6/76]) and AT-negative (6.9% [5/72] p = 0.6574) patients (total n = 148). The ICH-neurologic complication rate was similar for warfarin-positive (10.9% [5/46]) and AT-negative (6.9% [5/72] p = 0.5089) patients (total n = 118).

The ICH-neurologic complication rate was substantially increased when admission major neurologic dysfunction was present (63.2% [12/19]), compared to absent (2.2% [4/179]; RR = 28.3; p<0.001). The ICH-neurologic complication rate was substantially increased when ICH was present (19.4% [14/72]), compared to absent (1.6% [2/126]; RR = 12.3; p<0.001). Multivariate logistic regression analysis indicated that ICH-neurologic complications were independently associated with admission major neurologic dysfunction (p<0.001) and ICH (p = 0.0218), but not AT status (p = 0.8953). ICH-neurologic complications were independently associated with admission major neurologic dysfunction (p<0.001) and ICH (p = 0.0202), but not with platelet inhibitor-status (p = 0.7055). ICH-neurologic complications were independently associated with admission major neurologic dysfunction (p<0.001) and ICH (p = 0.0209), but not with warfarin-status (p = 0.7219). In the 72 patients with ICH, the ICH-neurologic complication rate was similar for the AT-negative (17.4% [4/23]) and AT-positive (20.4% [10/49]; p = 1.0) groups.

Of the 186 patients discharge alive from the trauma center, only one patient was unable to be adequately categorized according to the GOS classification. Of the 185 patients, a 3-month bad-outcome occurred in 18.9% (35/185) and a good-outcome in 81.1% (150/185). The 3-month bad-outcome rate was similar for the AT-negative (20.9% [14/67]) and AT-positive (17.8% [21/118]; p = 0.6050) patients. Univariate analysis showed that 3-month bad-outcome had a positive association with Injury Severity Score (p = 0.0130) and number of preinjury medical conditions (p = 0.0047) and an inverse association with admission GCS (p<0.0001). Multivariate analysis demonstrated that bad-outcome was independently associated with number of preinjury medical conditions (p = 0.0159) and admission GCS (p = 0.0088). Another analysis showed that bad-outcome was independently associated with number of preinjury medical conditions (p = 0.0098) and Injury Severity Score (p = 0.0632).

A summary of outcomes and their relationships with AT status and admission major neurologic dysfunction status is displayed in **Table 7**. The table demonstrates that admission major neurologic dysfunction status correlated with ICH, head AIS, ICH-neurologic complications, and GOS outcome. The table also indicates that AT status had no association with admission major neurologic dysfunction, ICH, head AIS, ICH-neurologic complications, or GOS outcome.

**Table 7:** Outcomes According to Antithrombotic and Admission Major Neurologic Dysfunction Status.

	AT (−)	AT (+)	P-value	AMND (−)	AMND (+)	P-value
**Number**	**72**	**126**	—	**179**	**19**	—
**AMND**	8.3%	10.3%	**0.6484**	—	—	—
**ICH**	31.9%	38.9%	**0.3285**	31.8%	79.0%	**<0.001**
**Head AIS**	1.9±1.6	1.9±1.6	**0.9509**	1.7±1.5	3.6±1.3	**<0.001**
**ICH Complication**	1.4%	5.6%	**0.2623**	0.0%	42.1%	**<0.001**
**Neurologic Complication**	6.9%	6.4%	**0.8708**	2.2%	47.4%	**<0.001**
**ICH-Neurologic Complication**	6.9%	8.7%	**0.6574**	2.2%	63.2%	**<0.001**
**Bad 3-Month Outcome**	20.9%	17.8%	**0.6050**	16.5%	66.7%	**0.0017**

AT, antithrombotic; (−), negative; (+), positive; AMND, Admission Major Neurologic Dysfunction; ICH, intracranial hemorrhage; AIS, Abbreviated Injury Scale score.

### Coagulation interventions

Coagulation interventions occurred in 28/198 (14.1%) select ICH-positive patients, who were AT-positive. Of the ICH-positive warfarin patients receiving coagulation intervention, all 13 received plasma transfusion and 12 Vitamin K. The mean time from initial therapeutic INR until the INR decreased to <2.0 was 10.6 hours. In ICH-positive patients receiving desmopressin and/or platelet transfusion, 16/18 had preinjury ASA and 3/18 had preinjury clopidogrel. The admission major neurologic dysfunction rate was greater in the 28 patients undergoing coagulation intervention (28.6%), compared to 170 without intervention (6.5%; p<0.001). Using multivariate logistic regression analysis, ICH-neurologic complication was independently associated with admission major neurologic dysfunction (p<0.001) and ICH (p = 0.0216), but not with AT-positive status (p = 0.9966) or coagulation intervention (p = 0.4160).

## Discussion

### Intracranial hemorrhage and antithrombotic agents

The current study focused on patients with documented signs of external head trauma or acute cervical spine injury to demonstrate that trauma to the craniofacial region existed, thus creating a risk for ICH. The similarity of the head trauma risk conditions for the AT-negative and AT-positive patients indicates that patient risk for developing an ICH is comparable. The coagulation studies demonstrate that most AT-positive patients had an objective measurable effect and therefore were taking their medications. The AT-positive and AT-negative ICH rates were similar, suggesting AT agents do not impede effective hemostasis. Further, there was no statistically significant variation according to the specific AT agent. The Injury Severity Score and AIS values indicate that the AT-positive and AT-negative patients had similar intracranial and extra-cranial injuries. Because head AIS values largely reflect the presence and magnitude of ICH, the similarity of these values for the AT-positive and AT-negative patients supports the comparable ICH rates, as determined by the investigator's review of each patient's brain CT. The similarity of AT-negative and AT-positive head AIS values in patients with ICH further demonstrates that AT agents are not associated with adverse TBI outcomes. Another study of elderly patients, sustaining an acute fall, has also demonstrated that there was no increased ICH rate with either warfarin, ASA, or clopidogrel [26].

The similarity of standard coagulation test, thromboelastography, and platelet mapping results for ICH-negative and ICH-positive patients indicates that a mechanism other than altered hemostasis is responsible for developing ICH. In addition, the relevant coagulation test results within each of the ASA, clopidogrel, and warfarin groups were comparable for ICH-negative and ICH-positive cohorts. Other investigators have found similar warfarin-associated ICH rates of 23.2% [29] and 26.6% [11] with external head trauma.

### Intracranial hemorrhage and preinjury brain atrophy

The current study demonstrates that preinjury composite brain atrophy is associated with the occurrence of acute post-traumatic brain injury. The higher atrophy rate in the platelet inhibitor-positive patients is likely to have contributed to its insignificantly higher ICH rate, when compared to AT-negative patients. This is strongly suggested by the multivariate analysis that showed ICH was associated with preinjury composite atrophy, but clearly not related to platelet inhibitor status. The other multivariate analyses also compellingly indicate that ICH correlates with brain atrophy, but not with AT agent status.

As age progressed, brain parenchyma width decreased and ventricular width, ventricular-to-brain parenchymal width, and cortical atrophy increased. At least six investigations of non-demented patients indicate that with advancing age, ventricular or intracranial CSF volume increases [30]–[34] and brain volume decreases [30]–[32], [34], [35]. A recent investigation used axial CT images and found that lateral ventricular volume-to-total brain volume markedly increased with aging [33]. Because multiple atrophy estimates in the current study correlate with age, this supports validity of the CT scoring. Also, the correlation of cortical atrophy with lateral ventricular width further enhances internal validity of the scoring methodology. Matsumae et al. concluded that “expansion of CSF volume with age provides a good index of brain shrinkage” [35]. ICH correlated with increased lateral ventricular body width, cortical sulcus atrophy, and composite brain atrophy. These new findings indicate that preinjury brain atrophy is a factor influencing the development of ICH, following head trauma.

Apropos to the current study, we found an interesting study by Ronty et al. that describes the use of linear CT measurements, that were related to brain atrophy [36]. These measures included linear assessment of the lateral ventricles, brain mass width, and cortical sulci. The study demonstrated that as brain atrophy volume increased, there were concomitant changes in these linear dimensions.

As stated earlier, speculation exists that preinjury cerebral atrophy fosters the development of acute post-traumatic ICH; however, we have been unable to find credible evidence to support this notion. The citation in one publication [27] referenced a textbook by Jennett and Teasdale [37]. Jennett and Teasdale, two prolific and revered neurosurgical investigators in TBI, provided no objective evidence to support their statement. In essence, the relationship between cerebral atrophy and intracranial hemorrhage, heretofore, is derived from expert opinion. Although traumatic ICH and acute CT findings increase with age [23], [38], as does cerebral atrophy, a direct association between preinjury atrophy and acute ICH has not been established. The current study is the first to objectively document that acute post-traumatic ICH is associated with preinjury cerebral atrophy.

### ICH-Neurologic complications and 3-month outcomes

Admission major neurologic dysfunction rates were similar for the AT-negative and AT-positive patients. A study of trauma patients by Mina et al. also found that warfarin did not decrease neurologic-function in patients with ICH [11]. These observations provide additional evidence that AT agents do not impact brain-injury. An ICH-neurologic complication was likely to be found in patients with major neurologic dysfunction on admission or with the presence of ICH. Importantly, none of the three conditions had an association with the preinjury use of AT agents. Other investigators have also demonstrated that AT agents are not associated with ICH progression [19], [20]. These findings mitigate the notion that AT agents substantially attenuate hemostatic function.

Accordingly, it is imperative to determine discriminate clinical findings that more focally portend the subset that will develop major complications. Apropos, the ICH-neurologic complication rate was substantially greater with admission major neurologic dysfunction, when compared to its absence. Multivariate logistic regression analysis provides evidence that admission neurologic status and ICH, but not AT use, forecasts the probability of developing an ICH-neurologic complication. Other investigators have also found that mortality is not increased for brain-injury patients receiving preinjury AT agents [13]–[17]. Ivascu et al. also demonstrated that presenting neurologic-function was predictive of death in trauma patients with ICH and receiving ASA and/or clopidogrel [39]. Peck et al., in a study assessing the influence of AT agents on ICH outcomes, showed that admission neurologic-function had the most statistically significant association with mortality [16]. The investigation by Gaetani et al. also demonstrated that presence of ICH increased mortality in a study of AT agents and brain-injury [9].

A warfarin TBI study by Wojcik et al. [17], included in a recent meta-analysis [22], is of particular interest. It is one of the better quality studies in that 416 TBI warfarin patients were compared to 416 case-controlled, non-warfarin patients. Age, ISS, and admission GCS were virtually identical for the 2 groups, as was mortality, ICU LOS, and hospital LOS (p≥0.70). In concert with our study findings are the two recent meta-analyses indicating that ASA and clopidogrel do not increase TBI mortality [21].

The 3-month GOS scores indicated that AT-negative and AT-positive patients had similar outcomes. It seems reasonable that post-discharge functional outcomes might be affected by preinjury medical conditions and/or severity of acquired traumatic conditions. This is suggested by the associations of post-discharge outcomes with the Injury Severity Score, admission GCS, and number of preinjury medical conditions. Together, the data results indicate that the preinjury use of AT-agents do not alter outcomes in patients who have sustained traumatic injury to the head. Further, these findings are in-concert with the study observations that post-traumatic ICH rates and acute, hospital outcomes were not worse in AT-positive patients.

### Coagulation interventions

Coagulation interventions occurred in select ICH-positive and AT-positive patients, who tended to have admission major neurologic dysfunction. Multivariate logistic regression analysis indicated that adverse outcomes occurred with admission major neurologic dysfunction and ICH, but not with coagulation interventions. Other studies have also documented that coagulation interventions did not alter outcomes [39], [40].

It seems reasonable that any AT-positive patient with external signs of head trauma should undergo prompt brain CT scanning and coagulation function testing. For a patient with ICH and no major neurologic dysfunction, it appears sensible to closely monitor the patient and initially only treat with a supratherapeutic coagulopathy. When an AT-positive patient with ICH has admission major neurologic dysfunction, there is uncertainty as to whether a coagulation intervention will mitigate subsequent adverse outcomes; although coagulation intervention would seem to be reasonable. It is important to recognize that acute or sub-acute AT age withdrawal, with or without reversal, can lead to adverse thrombotic events [41], [42].

### Evidence hemostasis is effective with antithrombotic therapy

Multiple non-trauma-related investigations demonstrate that ASA and warfarin do not routinely mitigate effective hemostasis. In particular, substantial literature evidence exists to indicate that ASA is not associated with major bleeding complications for surgery [43]–[48] and other invasive procedures [45], [49]. The literature also demonstrates that warfarin does not routinely create increased bleeding with operative procedures [50]–[53] and other invasive interventions [49], [54]–[56]. These findings suggest that ASA and warfarin do not usually attenuate clinically-relevant functional hemostasis following tissue injury.

### Study Quality

Although the study is retrospective, the following evidence indicates that common study weaknesses have been mitigated. Virtually all the relevant data is available for all patients and the quality is typically reliable; however, results for a few variables were not accessible for a minority of patients. Much of the data results emanate from the trauma registry, where information is obtained from the Trauma History and Physical form, the Trauma Tertiary Survey form, the trauma service Advanced Practice Nurses' daily work-sheet documentation, operative records, radiography reports, and discharge summaries. The Trauma History and Physical form is a comprehensive database completed at the time of patient admission by the trauma resident and verified by the attending trauma surgeon. At 24 hours following admission, a comprehensive Trauma Tertiary Survey form is completed by a trauma service resident and confirmed by the attending trauma surgeon. Each day, an Advanced Practice Nurse updated a work-sheet that captures initial injuries, all preinjury medications, including AT agents, injuries with a delay in diagnosis, and complications. Further, the Advanced Practice Nurses met weekly with the surgical attending staff to discuss patient deaths and all complications. This provides an opportunity to clarify ongoing patient conditions and outcomes in the nurses' daily work-sheet. Accordingly, mechanism of injury, admission and discharge GCS, preinjury AT agent use, preinjury medical conditions, patient injuries, and complications are prospectively documented and subsequently stored in the trauma registry. From injury data, trauma registry personnel compute Abbreviated Injury Scale and Injury Severity Score values. On multiple occasions, American College of Surgeons' Trauma Quality Improvement Program personnel have assessed trauma registry data and found it to be accurate and reliable, with compliance at all levels. Further, the State of Ohio Department of Public Safety has also appraised the trauma registry data and found it to be reliable. ICH status, ICH progression, and preinjury atrophy measurements were made by the first-author after reviewing CT scans for the 198 study patients. As an attending trauma surgeon with board certification in surgical critical care and an extensive history of collaboration with neurosurgeons and neuroradiologists, the first-author has interpreted brain CT images for 30 years. The presence of slurred speech and severe agitation in admission GCS 13–15 patients was made by the first-author after interrogating the Trauma History and Physical form, the neurosurgical consultation note, and nursing notes. The Trauma Service has long-standing and comprehensive policies regarding assessment and management of ASA, clopidogrel, and warfarin patients with signs of external head trauma or altered neurologic function. These policies, in large part, dictated coagulation testing and considerations for coagulation interventions.

We believe that information bias, improper classification of AT-exposure or outcomes, is minimal, because the data quality is creditable. The 3-month GOS designation was assigned following an extensive review of the electronic medical record, where virtually all patients could be readily classified into a bad-outcome or good-outcome category. We also think that selection bias has been minimized by using the trauma registry to identify all patients who were age ≥60 and with signs of external head trauma or cervical spine injury. Minimal selection bias is supported by finding a similarity in the AT-negative and AT-positive patient Injury Severity Score, ICH-risk condition rates, extra-cranial injury severity, and admission major neurologic dysfunction rates.

### Conclusions

The similarity of AT-negative and AT-positive group major admission neurologic dysfunction rates, ICH rates, head AIS scores, hospital complication rates, and 3-month neurologic function rates indicate that AT-agents do not adversely affect brain injury outcomes in patients with signs of external head trauma. Current study outcomes and coagulation test results and the procedural intervention literature suggest that AT agents do not routinely impede effective post-injury hemostasis. It is likely that our study findings will stimulate trauma services to review, and potentially refine, policies regarding management of AT-positive patients with head trauma. Clearly, admission neurologic function is useful for forecasting adverse outcomes in patients with external head trauma, whether or not they receive a preinjury AT agent. For AT-positive patients with ICH, but without major neurologic dysfunction, we recommend close patient monitoring and coagulation interventions for those with supratherapeutic coagulopathy. When an AT-positive patient with ICH has admission major neurologic dysfunction, coagulation intervention seems reasonable; however, there is uncertainty as to whether this course of action will mitigate subsequent adverse outcomes. Considering the substantial activity in developing AT-drug reversal agents, it is imperative that criteria be created to stipulate which patients are likely to benefit from such interventions. The correlation of ICH with atrophy is the first objective evidence that acute post-traumatic ICH is associated with preinjury cerebral atrophy. It seems reasonable that radiologists should begin to assess the magnitude of brain atrophy in patients with an age ≥60 years. If the lateral ventricular body width is>30 mm and/or the cortical sulci score is 2 or 3, the radiologist should indicate that significant brain atrophy is likely present. Accordingly, the treating physician should assess the patient using one of the standard fall-risk assessment tools. If the risk for falling is substantial, the healthcare provider should institute appropriate measures that mitigate the risk of falling.
